# The Effects of Single- or Mixed-Strain Fermentation of Red Bean Sourdough, with or without Wheat Bran, on Bread Making Performance and Its Potential Health Benefits in Mice Model

**DOI:** 10.3390/foods13172856

**Published:** 2024-09-09

**Authors:** Chengye Huang, Binle Zhang, Jing Huang, Youyi Liu, Cheng Chen, Jacob Ojobi Omedi, Li Liang, Zhongkai Zhou, Weining Huang, Ning Li

**Affiliations:** 1 State Key Laboratory of Food Science and Resources, Laboratory of Baking and Fermentation Science, Cereals/Sourdough and Nutritional Functionality Research, School of Food Science and Technology, Jiangnan University, Wuxi 214122, Chinaomedij@yahoo.com (J.O.O.); liliang@jiangnan.edu.cn (L.L.); 2 Deapartment of Food Engineering, Zhangzhou Institute of Technology, Zhangzhou 363000, China; 3 Wuxi School of Medicine, Jiangnan University, Wuxi 214122, China; 4 College of Food Science, Shihezi University, Shihezi 832003, China; 5 Guangzhou Puratos Food Co., Ltd., Guangzhou 511400, China; lning@puratos.com

**Keywords:** sourdough, single- and mixed-strains fermentation, red bean, wheat bran, bread quality, health benefits

## Abstract

The effects of single- (*Lactobacillus fermentum*) or mixed-strain (*Lactobacillus fermentum*, *Kluyveromyces marxianus*) fermentation of red bean with or without wheat bran on sourdough bread quality and nutritional aspects were investigated. The results showed that, compared to unfermented controls, the tannins, phytic acid, and trypsin inhibitor levels were significantly reduced, whereas the phytochemical (TPC, TFC, and gallic acid) and soluble dietary fiber were increased in sourdough. Meanwhile, more outstanding changes were obtained in sourdough following a mixed-strain than single-strain fermentation, which might be associated with its corresponding β-glucosidase, feruloyl esterase, and phytase activities. An increased specific volume, reduced crumb firmness, and greater sensory evaluation of bread was achieved after mixed-strain fermentation. Moreover, diets containing sourdough, especially those prepared with mixed-strain-fermented red bean with wheat bran, significantly decreased serum pro-inflammatory cytokines levels, and improved the lipid profile, HDL/LDL ratio, glucose tolerance, and insulin sensitivity of mice. Moreover, gut microbiota diversity increased towards beneficial genera (e.g., *Bifidobacterium*), accompanied with a greater increase in short-chain fatty acid production in mice fed on sourdough-based bread diets compared to their controls and white bread. In conclusion, mixed-strain fermentation’s synergistic effect on high fiber-legume substrate improved the baking, sensory quality, and prebiotic effect of bread, leading to potential health benefits in mice.

## 1. Introduction

Sourdough fermentation, an ancient bioprocessing technology, has commonly been used to improve the nutritional, functional, and sensory aspects of bread [1,2,3]. In legume ingredients, sourdough fermentation significantly reduced the anti-nutritional factors (ANFs) and off-flavors which improved their use as ingredients in wheat-composite sourdough bread systems. For instance, the baking quality and overall acceptability of wheat-soybean bread was improved after soybean flour fermentation using *Pedicoccus pentosaceus* and *Lactobacillus plantarum* strains [4]. In another study, incorporation of *Weissella confusa* fermented red bean sourdough improved the dough rheo-fermentation properties, and the quality and aroma of wheat–red bean bread [5]. Moreover, the content of ANFs of trypsin inhibitor and phytate were reduced in legume sourdoughs and the resulted wheat–legume-based sourdough bread [6]. On the other hand, fermentation of high fiber ingredients such as wheat bran reduced the insoluble dietary fiber (IDF) content through its increased solubilization to soluble dietary fiber (SDF). Pre-fermentation of wheat bran by *Kluyveromyces marxianus* enhanced wheat bran functionality in bread making [7]. Specifically, the changes attributed to sourdough were linked to the type of microbial starters used; their metabolism of different substrates release bioactive metabolites aided by insitu-produced enzymes during the fermentation process [3,8]. 

Published studies have reported that sourdough fermentation using mixed strains such as lactic acid bacteria (LAB) and yeast, provided more effective and consistent outcomes than single-strain fermentation in the development of functionally enriched baked products [9]. This was attributed to a synergistic interactive effect between LAB and yeast strains, where LAB provided energy sources from sugars released for yeast growth and yeast provided nutrients such as vitamins and amino acids for LAB growth during fermentation [10,11]. Subsequently, compared to single yeast or LAB-strain-fermented sourdoughs, a significantly higher increase in bioactive metabolites and a reduction of ANFs were found in mixed yeast and LAB-strain-fermented sourdoughs and the subsequent end product [12,13]. In a mixed-strain (*Lactobacillus plantarum* or *Pediococcus pentosaceus* and *K. marxianus*) fermented white kidney bean sourdough, bioactive peptides increased and ANFs reduced, whereas the resulted sourdough bread had significantly increased resistant starch, slowly digestible starch, and protein digestibility [6]. Moreover, intake of this sourdough bread increased gut microbiota diversity, enhanced anti-inflammatory effects, and improved glucose tolerance compared to white kidney bean bread in healthy mice. However, few published studies on the effect of LAB and yeast-strain fermentation on the nutritional and functional properties of red bean flours supplemented with wheat bran have been reported. 

Furthermore, changes in consumer tastes and preferences have fueled development of functional and bioactive-ingredient-enriched food products with potential physiological effect when consumed [14]. Red beans contain high-quality proteins, fibers, and phenolic compounds which have potential nutritional and health-promoting effects in functional food products [15]. On the other hand, wheat bran in functional foods could enhance the antidiabetic properties and prebiotic effects on gut health [16,17]. However, studies have shown that the addition of red bean flour and wheat bran in wheat dough/bread systems led to poor product quality and decreased consumer product acceptability. This was attributed to the presence of off-flavors and ANFs in legumes and the high IDF content in wheat bran which, collectively, diluted gluten proteins, increased water competition with gluten proteins during hydration, and disrupted gluten network formation [15,16,17,18,19]. To counter these shortcomings, the use of sourdough fermentation to improve legume and wheat bran applicability to enhance the technological, nutritional, and functional properties and overall acceptability of wheat-composite baked goods may be useful [20,21]. Furthermore, the impact of the co-existence of red bean and wheat bran as substrate in a mixed-strains sourdough system in terms of physicochemical and nutritional aspects is far from investigated.

Therefore, the objective of this study was to investigate the effect of single (*Lactobacillus fermentum*) or mixed (*Lactobacillus fermentum* and *Kluyveromyces marxianus*) strain sourdough fermentation on the physicochemical characteristics such as anti-nutritional factors, total phenolic and flavonoid content, and dietary fiber composition of red bean with or without wheat bran flour. Changes in key enzymatic activities such as β-glucosidase, feruloyl esterase, and phytase during sourdough fermentation were reported. The quality of the corresponding breads based on specific volume, textural profile characteristic, and sensory evaluation were also reported. Furthermore, changes in lipid profile, glucose tolerance, insulin resistance, pro-inflammatory cytokine, short-chain fatty acids, and gut microbiota composition in healthy mice after 28 days of consuming the sourdough breads were also evaluated.

## 2. Materials and Methods

### 2.1. Materials

High-gluten wheat flour was purchased from Xinxiang Liangrun Whole Grain Food Co., Ltd. (Xinxiang, China). Red bean (whole seed) flour was purchased from Chengwu County Grain and Edible Oil Storage Market Department in Shandong (Heze, China), and wheat bran flour was obtained from Henan Jinyuan Grain and Oil Co., Ltd. (Jinyuan, China). External standards for phenolic acid (ferulic acid, gallic acid, protocatechuic acid, chlorogenic acid, caffeic acid, and ρ-coumaric) (purity ≥ 99.5%) and short-chain fatty acid (purity ≥ 99.5%) were purchased from China National Pharmaceutical Group Chemical Reagent Co., Ltd (Shanghai, China). Other chemicals used were of analytical grade.

### 2.2. Sourdough Preparation of Red Bean Supplemented with Wheat Bran via Either Single or Mixed-Strains Fermentation

#### 2.2.1. Preparation of LAB and Yeast Inoculum

LAB strain *Lactobacillus fermentum* YC’22, obtained from the Laboratory of Baking and Fermentation Science, Cereals/Sourdough and Ingredient Functionality Research, Jiangnan University, was cultured at 37 °C in MRS for 24 h, followed by centrifugation at 8000× *g* for 8 min at 4 °C, then washed twice with sterile saline solution to obtain the LAB (10^7^ CFU/g) inoculum. Yeast strain *Kluyveromyces marxianus*, purchased from Shanghai Ye Yan Biological Technology Co., Ltd. (Shanghai, China), was cultured at 30 °C YPD broth for 24 h, then centrifuged (8000× *g* for 8 min at 4 °C) and washed twice with sterile saline solution to obtain the and yeast (10^7^ CFU/g) inoculum. 

#### 2.2.2. Single- and Mixed-Strains Sourdough Fermentation

For single-strain sourdough fermentation, LAB (10^7^ CFU/g) inoculum was resuspended in 10 mL of sterile water and inoculated into 100 g of either red bean flour or red bean with added wheat bran (5:4, *w*/*w*) substrate with a dough yield (DY) [(amount of flour + amount of water)/amount of flour) × 100] of 300 (corresponding to 190 mL of water and 100 g of flour). For mixed-strain sourdough fermentation, a suspension of LAB (10^7^ CFU/g) and yeast (10^7^ CFU/g) (ratio 1:1) were added to 100 g of the same substrate (red bean flour or red bean flour with added wheat bran) with a DY of 300 (corresponding to 190 mL of water and 100 g of flour). The inoculated mixtures were sealed and fermented at 30 °C for 24 h to obtain the single- and mixed-strain-fermented sourdough for bread preparation. 

### 2.3. Physicochemical Analysis of Single- and Mixed-Fermented Sourdough of Red Bean Following Wheat Bran Supplementation

#### 2.3.1. pH and Total Titratable Acidity (TTA) of Sourdough Following Different Fermentation

The pH and TTA of the samples were measured before and after sourdough fermentation. Briefly, 10 g of samples were homogenized with 90 mL distilled water, and the pH was measured using a pH meter (FE-20, Mettler Toledo, Shanghai, China). The TTA was expressed as mL of 0.1 N NaOH needed to achieve a pH of 8.5.

#### 2.3.2. Trypsin Inhibitor Activity (TIA), Phytic Acid (PA), and Condensed Tannin Content

The TIA in freeze-dried sourdough samples was determined as described by Klomklao and Benjakul using the Nα-benzoyl-L-arginine 4-nitroanilide hydrochloride (BApNA) assay [22]. Briefly, 200 mL of freeze-dried sample was extracted overnight with 0.15 M phosphate buffer (pH 8.1) at 4 °C. This was followed by centrifugation (4000× *g*, 15 min, 15 °C), addition of 200 mL porcine pancreatin (2 g/L), and incubation at 37 °C for 15 min. A preheated mixture containing 800 mL of BApNA and 200 mL of distilled water was added and incubated with shaking for 10 min. The reaction was terminated by adding 300 mL of acetic acid. The reaction mixture was centrifuged (8000× *g*, 5 min, 4 °C), and the residual trypsin activity was measured at 410 nm. One unit of TIA was defined as the amount of inhibitor required to reduce enzyme activity by one unit under the assay conditions, and the results were expressed as units of trypsin inhibition per gram of sample (U/g).

The PA content in freeze-dried sourdough was determined according to the method described by Buddrick et al. [23]. Absorbance was measured at 519 nm using a UV spectrophotometer (TU 1810, Beijing, China) with distilled water used as the blank. A standard curve was constructed using dilutions in the stock solutions of PA standard dissolved in hydrochloric acid.

Condensed tannins in freeze-dried sourdough were determined using the vanillin method described by Broadhurst and Jones [24]. Briefly, 1 g of freeze-dried sourdough was extracted with 10 mL of hydrochloric acid and methanol (1:100, *v*/*v*) by shaking at room temperature for 2.5 h (in darkness). This was followed by centrifugation (4000× *g*, 20 min) to obtain the supernatant for analysis. A standard curve was constructed using catechin, and the results were expressed as catechin equivalents.

#### 2.3.3. Total Phenolic, Total Flavonoid Content, and Phenolic Acid Composition of Sourdough Following Different Fermentation 

Extracts from samples were prepared by adding 10 mL of methanol to 2 g freeze-dried sourdough sample at 25 °C and 200 rpm for 24 h. The residue was subsequently re-extracted with methanol after filtration and this process was repeated three times. All extracts were filtered through a 0.22 μm filter, adjusted to 10mL with methanol, and stored for subsequent analysis. 

The total phenolic content (TPC) in extracts was determined following the Folin–Ciocalteu method described by [25]. Absorbance was measured at 760 nm. Gallic acid (GA) was used as the standard, distilled water was the blank, and the TPC was expressed in terms of gallic acid equivalents (mg GAE/g). The total flavonoid content (TFC) in extracts was determined as described by [26]. The TFC was expressed as rutin equivalents (mg RE/g).

Phenolic acid composition in the methanol extracts was quantified using the reverse-phase HPLC method as previously described by Omedi et al. [27]. Analysis was carried out using the Waters HPLC system consisting of a Waters 1525 Binary HPLC pump, Waters 2707 Autosampler, and Waters 2489 UV–vis detector (Waters Corporation, Milford, MA, USA). Chromatographic separation was performed on a SYMMETRY C18 (5 µm, 4.6 × 250 mm) column. Elution was carried out with mobile phase (A) 1% aqueous acetic acid solution; mobile phase (B) acetonitrile. Flow rate was 0.7 mL/min, the UV/Vis detector used 310 nm wavelength, and 35 °C was the column temperature. Elution gradient: 90% A (0–27 min), 10–40% B (28 min), 40–60% B (28–39 min), 60–90% B (39–50 min), then back to initial condition 90% A in 55 min. This was allowed to run for another 5 min, before the injection of another sample. Total analysis time per sample was 60 min. Methanol extracts were filtered (0.22 µm filter) prior to HPLC injection and 20 µL of sample was injected. Data acquisition and integration was performed using the Empower (version 3.0) software package. The identification of phenolic acids in extracts was performed using external standards analyzed under similar conditions.

#### 2.3.4. Dietary Fiber Composition of Sourdough Following Different Fermentation

Dietary fiber composition, including total dietary fiber (TDF), soluble dietary fiber (SDF), and insoluble dietary fiber (IDF) were determined using AACC method 32-07. 

### 2.4. Changes in β-Glucosidase, Phytase, and Feruloyl Esterase Enzyme Activities during Sourdough Fermentation

To determine changes in enzyme activities, 1 g samples were collected at 0, 4, 8, 12, 16, 20, and 24 h periods during the sourdough fermentation process. The collected sample was mixed with 20 mL of deionized water, and centrifuged (10,000× *g*, 4 °C, 15 min) to obtain supernatant which was used as an enzyme solution to determine the enzyme activities. 

β-glucosidase activity was assayed by measuring the amount of p-nitrophenol (p-NP) released from the substrate 4-nitrophenyl β-D-glucopyranoside (p-NPG) as described by Liang et al. [8]. Briefly, to 100 μL of enzyme solution, 1.8 mL of 0.05 M sodium acetate buffer (pH 5) was added and incubated at 37 °C for 5 min. Then, 100 μL of p-NPG was added and the mixture was further incubated at 37 °C for 10 min. The reaction was stopped by adding 1 mL of 1 M Na_2_CO_3_ and the absorbance was measured at 400 nm. A standard curve using β-glucosidase enzyme (Shanghai Yuanye Biological Technology Co., Ltd., Shanghai, China) was used to determine enzyme activity. Enzyme unit was defined as the amount of enzyme required to catalyze the production of 1 μmol of p-NP per minute under the same conditions.

Feruloyl esterase activity was determined using the method described by Liang et al. [8] with some modifications. Briefly, 500 μL of enzyme solution was mixed with 500 μL of 2 mg/mL methyl ferulate buffer solution, and incubated at 45 °C for 10 min. After, 1 mL of 0.01 mol/L glycine (pH = 10) was added to stop the reaction. The absorbance was measured at 335 nm. Enzyme activity unit was defined as the amount of enzyme required to release 1 μmol of ferulic acid by hydrolyzing ferulic acid methyl ester per minute under the same conditions.

Phytase activity was determined using the method described by Fekri et al. [28] with some modifications. Briefly, 0.2 mL of enzyme solution was mixed with 1.8 mL of sodium acetate buffer and incubated at 37 °C for 5 min. Afterward, 4 mL of 7.5 mmol/L sodium phytate solution was added and incubated at 37 °C for 30 min. The reaction was terminated and the color development was induced by adding a termination and chromogenic solution (a mixture of 30% nitric acid solution, 100 g/L ammonium molybdate, and 2.35 g/L ammonium metavanadate solution at a ratio of 2:1:1). Absorbance was measured at 415 nm. Enzyme activity was defined as the amount of enzyme capable of releasing 1 μmol of inorganic phosphate per min at 37 °C.

### 2.5. Sourdough Bread Preparation Following Either Single- or Mixed-Strains Fermentation 

In this study, 7 types of bread were studied, including, (1) wheat bread (WB), (2) red bean bread (RB), (3) red bean–wheat bran bread (RWB), (4) red bean sourdough bread fermented by *L. fermentum* (RBY), (5) red bean sourdough bread fermented by *L. fermentum* and *K. marxianus* (RBYK), (6) red bean–wheat bran sourdough bread fermented by *L. fermentum* (RWBY), and (7) red bean–wheat bran sourdough bread fermented by *L. fermentum* and *K. marxianus* (RWBYK). The recipe for each bread was shown in Table S1. For bread preparation, briefly, all weighed ingredients, except margarine, were mixed in a spiral mixer (Sinmag, Wuxi, China) at slow speed for 3 min then fast speed 1 min. Shortening was then added and mixed at slow speed for 1 min, followed by high speed for 3 min. The dough was then covered with polyethylene film and rested (10 min) at room temperature. Dough was then divided (90 g piece), rounded, shaped, transferred into baking pans, then proofed (Sinmag, Wuxi, China) (90 min, 38 °C, 85% RH). This was followed by baking in a preheated oven (Sinmag, Wuxi, China) (top: 170 °C and bottom: 220 °C) for 21 min. After baking, the bread was cooled for 2 h at room temperature. A portion was used for further analysis, while the rest was dried (40 °C, 9 h) and ground into a powder to prepare the feed diet for animal trials. 

### 2.6. Physicochemical Analysis of Sourdough Bread Prepared with Either Single- or Mixed-Strains Fermentation

#### 2.6.1. Specific Volume

The specific volume of bread was measured 2 h after baking using the rapeseed displacement method. Specific volume (mL/g) was defined as the ratio of bread volume (mL) to mass (g).

#### 2.6.2. Textural Profile Analysis

After cooling, the bread was sliced into thin slices of 10 mm using a slicer. Two middle slices were selected and used to determine the textural profile of bread using a Texture Pro CT V 1.4 Build 17 (Brookfield Engineering Laboratory, Middleboro, MA, USA) in the texture profile analysis (TPA) test mode, consisting of a double compression test and equipped with an aluminum 36mm diameter cylindrical probe. The testing conditions were as follows: probe model P/36, probe movement speed before testing at 1.0 mm/s, probe movement speed during compression testing at 3.0 mm/s, probe movement speed after compression completion at 3.0 mm/s, compression strain at 50%, induction force at 5 g, compression time interval of 1 s between two compressions. The parameters reported on were firmness and chewiness. 

#### 2.6.3. Sensory Evaluation

Twenty semi-trained individuals (10 males, 10 females) from the School of Food Science and Technology, Jiangnan University (China), evaluated the sensory attributes, including the shape, color, flavor, texture, mouthfeel, and overall acceptability of bread using the nine-point hedonic scale. Scores ranging from 1 to 9 (1: dislike extremely, 2: dislike very much, 3: dislike moderately, 4: dislike slightly, 5: neither like or dislike, 6: like slightly, 7: like moderately, 8: like very much, and 9: like extremely) were assigned to indicate the degree of preference for various bread attributes. Each bread sample was sliced, randomly coded, and given to the panelists at least 2 h after baking. The panelists were instructed to rinse their mouths with water between samples to minimize any residual effects.

### 2.7. Customized Bread Diets and Animal Study Design

#### 2.7.1. Preparation of Customized Bread Diet

The bread powder (Section 2.5) was mixed with standard mice diet (AIN-93G) in a 1:1 ratio to prepare customized bread diets. To meet specific pathogen-free (SPF) standards, the mixture was processed into pellets, irradiated, and then vacuum-packed by Anhui Kui Bu Qian Li Biotechnology Co., Ltd. (Xuancheng, China) (nutritional information of the diet is shown in Table S2).

#### 2.7.2. Animal Experimental Design

A total of 42 male 8-week-old C57BL/6J mice purchased from Vital River Laboratories (Beijing, China) were kept in the SPF animal facility with a 12 h light/12 h dark cycle and maintained at a controlled temperature (22 ± 2 °C). During a seven-day acclimatization period, mice were provided ad libitum access to standard diet (AIN-93G) and water for 6 days, followed by a 24 h fast with ad libitum water access on the seventh day. After acclimatization, mice were randomly divided into 7 groups (*n* = 6), with each group placed in separate cages. Subsequently, mice in separate cages were fed their respective diets ad libitum for 28 days with free access to water. On the 28th day, following an overnight fast, blood samples were collected from the orbital venous plexus of each mouse’s eye, and then the mice were killed by decapitation. The animals were maintained and handled in accordance with the guidelines of the ethics committee involving use of animals in Jiangnan University which approved this study (JN.No20230915c0841119[382]).

### 2.8. Potential Health Benefits of Intake of the Customized Bread Diets on Mice

#### 2.8.1. Anti-Inflammatory Effect Based on Pro-Inflammatory Content in Mice 

The levels of pro-inflammatory cytokines, including tumor necrosis factor-alpha (TNF-α), interleukin-1 beta (IL-1β), interleukin-6 (IL-6), and endotoxin lipopolysaccharide (LPS) in serum samples was determined using ELISA kits (Nanjing SenBeiJia Bio-Technology Co., Ltd., Nanjing, China). 

#### 2.8.2. Serum Lipid Profile in Mice 

The blood lipid profile in serum of mice, including triglyceride (TG), total cholesterol (TC), high-density lipoprotein (HDL) cholesterol, and low-density lipoprotein (LDL) cholesterol, was determined using ELISA kits (Nanjing SenBeiJia Bio-Technology Co., Ltd.). 

#### 2.8.3. Oral Glucose Tolerance Test (OGTT)

The OGTT was determined on the final day of the experiment. On the 27th day, mice were fasted overnight, followed by intraperitoneal injection of 0.2 mL glucose solution (10% in saline). The baseline glucose values (0 min) were recorded before the gavage administration of glucose solution. After the injection, blood was collected via a small cut made at the tip of each mouse’ tail. A small drop of blood from the tail vein was quickly absorbed into a test strip for glucose determination using a glucometer (ACCU-CHEK Active, Corydon, IN, USA). The blood from the tail was measured at regular intervals of 30, 60, 90, and 120 min after injection of glucose. 

#### 2.8.4. Insulin Resistance (HOMA-IR) Analysis

Fasting insulin level was determined using the mouse ELISA kit (SenBeiJia Biological Technology Co., Ltd., Nanjing, China). The homeostasis model assessment-insulin resistance (HOMA-IR) was calculated to determine insulin sensitivity using the following formula:

HOMA-IR = [(Fasting insulin × Fasting blood glucose)/22.5].(1)

### 2.9. Gut Microbiota Composition 

Fecal samples collected on the 27th day of the experiment were used to analyze the changes in gut microbiota composition. Microbial genomic DNA was extracted from frozen fecal samples using the TransGen AP 221-02: TransStart FastPfu DNA polymerase kit (TransGen Biotech, Beijing, China). The V3 + V4 region of the 16S rRNA gene was amplified by PCR and sequenced using the Illumina HiSeq 2500 PE 300 platform. 16S rRNA gene sequencing was performed by Beijing Novogene Bioinformatics Technology Co., Ltd. (Beijing, China).

### 2.10. Short-Chain Fatty Acid (SCFA) Content in Feces of Mice

The content of SCFAs in feces was determined according to the method described by [29] with some modifications. Briefly, 50 mg fecal samples were homogenized in 1 mL Milli-Q water for 3 min, followed by a 10 min incubation at room temperature and a centrifugation at 12,000× *g* for 10 min at 4 °C. The supernatant was then filtered through a 0.45 μm cellulose acetate filter, and 400 μL of the filtered supernatant was mixed with 100 μL of a 50 μmol/mL internal standard (2-ethylbutyric acid) solution, 10 μL of formic acid, and 490 μL of Milli-Q water in a polypropylene vial. The mixture was then centrifuged at 12,000× *g* for 15 min at 4 °C, and 700 μL of the supernatant was collected for SCFA analysis. The SCFAs content was determined using GC (Clarus 680 Gas Chromatography, PerkinElmer, Inc., Waltham, MA, USA), equipped with a HP-INNOWAX column (30 m × 0.250 mm × 0.25 μm, Agilent Technologies Inc., Santa Clara, CA, USA), with helium used as the mobile phase (flow rate: 1 mL min^−1^).

### 2.11. Statistical Analysis

Results from at least three independent measurements were presented as the mean value. Data were compared by one way analysis of variance (ANOVA) using SPSS 26.0 (IBM, Armonk, New York, NY, USA) and a comparison between groups was conducted via Duncan’s test. Significant differences were considered when *p* < 0.05. Additionally, Origin 8.0 (OriginLab, Northampton, MA, USA) and R language 3.5.0 were utilized. A Pearson correlation test was performed to explore associations among dough/bread parameters, gut microbiota, cytokine content, and potential health benefits in mice. 

## 3. Results and Discussion

### 3.1. Physicochemical Characteristics of Sourdough via Either Single- or Mixed-Strains Fermentation with Supplementation of Wheat Bran

#### 3.1.1. Impact of Single- or Mixed-Strains Fermentation on the Changes in pH and TTA of Sourdough

The changes in pH and TTA of sourdough samples are presented in Table 1. The results showed that the pH was 6.46 and 6.39 in RB and RWB controls, respectively. After fermentation, pH values decreased (*p* < 0.05) in the range of 4.23 to 4.37, with lower values observed in mixed- (RWBYK < RBYK) than single-strain (RWBY < RBY) fermented red bean flour with wheat bran sourdoughs. The TTA values were 4.52 and 4.48 in RB and RWB controls, but increased (*p* < 0.05) in the range 18.28 to 20.39 mL after fermentation. Higher TTA values were seen in mixed-strain- than single-strain-fermented red bean flour with wheat bran sourdoughs (Table 1). The pH and TTA changes after sourdough fermentation were in agreement with commonly reported trends in acidification parameters in several sourdough fermentation systems [2]. In sourdough studies, pH and TTA changes were associated with LAB and/or yeast cell growth which metabolized substrate nutrients to release pH-altering metabolites such as organic acids. Additionally, due to synergistic interactions during fermentation, the use of LAB and yeast in mixed-strain fermentation could significantly lower the pH and increase TTA of the sourdough [6,30]. In this study, the synergy in mixed-strain fermented red bean supplemented with wheat bran could have provided complimentary nutrition for better proliferation and metabolism of LAB and yeast which led to release of more pH-altering metabolites than in single-strain-fermented substrates. 

#### 3.1.2. Impact of Single- or Mixed-Strains Fermentation on the Changes in the Anti-Nutritional Factors (ANFs) Content of Sourdough

The content of ANFs, including phytic acid (PA), trypsin inhibitor activity (TIA), and condensed tannins in sourdoughs was shown in Table 1. Compared to their respective unfermented controls, sourdough fermentation generally decreased (*p* < 0.05) the ANF content in substrates. Higher decreases were observed in red bean supplemented with wheat bran after mixed-strain fermentation than single-strain fermentation. For instance, compared to RB and RWB, the PA decreased by −22.17, −23.90, −29.43, and −30.08%, and the TIA decreased by −41.66, −41.20, −45.92, and −45.23 in RBY, RBYK, RWBY, and RWBYK, respectively. Condensed tannins were lower in RWBY and RWBYK than RBY and RBYK; however, a higher decline rate was seen in RBY and RBYK (−40.17 to −41.17%) than in RWBY and RWBYK (−31.21 to −32.35%) compared to their controls. This was attributed to the higher condensed tannins content in the RB than RWB as the starting substrate (Table 1). Higher declines in the presence of wheat bran indicated that the additive effect of wheat bran could have increased the amount of ANFs, specifically phytates and trypsin inhibitors which were degraded more during mixed-strain than single-strain sourdough fermentation of red bean supplemented with wheat bran. Wheat bran is reported to contain bound metabolites such as phenolic acids and phytates which can be released through sourdough fermentation [31,32]. 

#### 3.1.3. Impact of Single- or Mixed-Strains Fermentation on the Changes in Key Enzymatic Activities during Sourdough Fermentation

**Figure 1:**
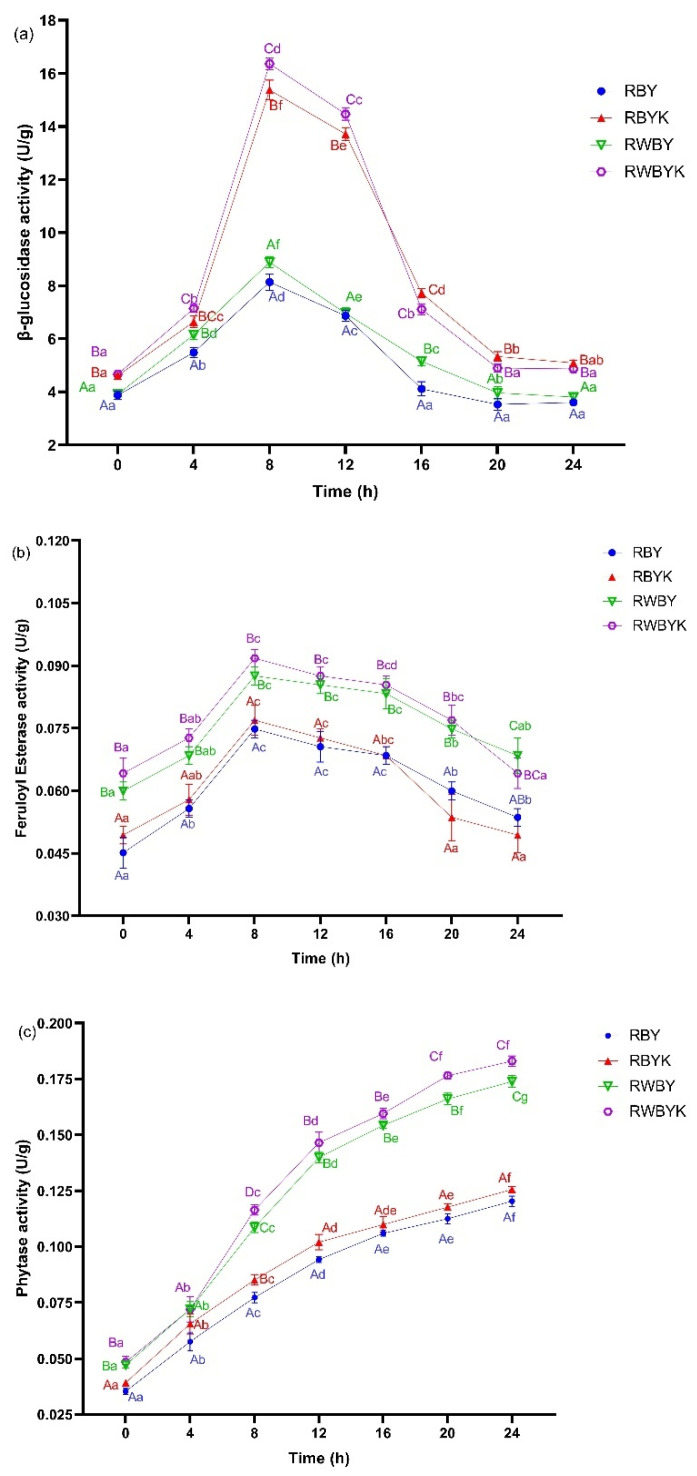
The changes in β-glucosidase (**a**), feruloyl esterase (**b**), and phytase (**c**) enzyme activity during single- and mixed-strain sourdough fermentation of red bean flour with or without wheat bran. RBY: red bean sourdough fermented by *L. fermentum*; RBYK: red bean sourdough fermented by *L. fermentum* and *K. marxianus*. RWBY: red bean–wheat bran sourdough fermented by *L. fermentum*. RWBYK: red bean–wheat bran sourdough fermented by *L. fermentum* and *K. marxianus*. Different lower-case and upper-case letters indicated significant differences at *p* < 0.05 for the same sample at different time of fermentation and different samples at the same time of fermentation, respectively.

Changes in β-glucosidase, feruloyl esterase, and phytase activities during sourdough fermentation of red bean supplemented with wheat bran are presented in Figure 1. Generally, all the three enzyme activities were detected during the sourdough fermentation process. For β-glucosidase, its activity was higher (*p* < 0.05) during mixed-strain (RWBYK > RBYK) than single-strain (RWBY > RBY) substrate (higher in red bean with wheat bran than without) fermentation (Figure 1a). Yeast *K. marxianus* are high β-glucosidase producers [7]; this could have led to the additionally higher β-glucosidase activities in RWBYK and RBYK than RWBY and RBY during mixed-strain sourdough fermentation. On the other hand, feruloyl esterase activity was significantly (*p* < 0.05) higher in mixed-fermented red bean with wheat bran (RWBYK > RWBY) than without (RBYK > RBY) substrate during sourdough fermentation (Figure 1b). Alteration of the optimum pH ideal for microbial-produced esterase enzyme can influence its activity during substrate fermentation [8]. In this study, the addition of wheat bran in red bean flour may have had a buffering effect on pH and maintained it within the optimum for the feruloyl esterase activity produced during sourdough fermentation (Table 1, Figure 1b). Similar to the feruloyl esterase, phytase activity was higher (*p* < 0.05) in red bean flour supplemented with wheat bran (RWBYK > RWBY) than without (RBYK > RBY) during sourdough fermentation (Figure 1c). Unlike the effect of pH on esterase activity, in addition to phytase produced by the starters during fermentation, sourdough acidification could have activated endogenous phytases in wheat bran leading to a significantly higher activity in RWBYK and RWBY than in RBYK and RBY during fermentation [33,34]. Overall, the in situ-produced enzymes during sourdough fermentation are responsible for diverse bio-transformations such as phytases for phytic acid degradation, esterase, and β-glucosidase for fiber solubilization which increase the nutritional, bioactive potential of sourdough and the resulted sourdough bread [2,3].

### 3.2. Bioactive Content of Single- or Mixed-Strains-Fermented Red Bean Supplemented with Wheat Bran Sourdough

The total phenolic content (TPC) and total flavonoid content (TFC) of sourdough samples are presented in Table 1. Compared to RB (4.02 mg GAE/g) and RWB (4.95 mg GAE/g), the TPC increased (*p* < 0.05) in all samples after sourdough fermentation. A higher increase was seen in mixed-strain than single-strain red bean with wheat bran (RWBYK (+44.90%) > RWBY (+33.77%)) than without (RBYK (+39.90%) > RBY (+30.11%)) sourdough samples. On the other hand, compared to RB (2.39 mg RE/g) and RWB (2.31 mg RE/g), the TFC increased (*p* < 0.05) in RBY (23.12%), RBYK (39.30%), RWBY (22.00%), and RWBYK (37.27%). In several published studies, the release of bound phytochemicals due to hydrolytic activity of in situ-produced enzymes during microbial fermentation increased the TPC and TFC of the fermented substrate [35,36]. As shown in Figure 1, enzyme activities (phytase, feruloyl esterase, and β-glucosidase) increased during sourdough fermentation, especially higher (*p* < 0.05) in mixed- than single-strain-fermented red bean with wheat bran than without sourdough. This could be vital in the release of bound phytochemicals which increased the TPC and TFC content of sourdough (Table 1). 

The phenolic acids, including gallic acid, protocatechuic acid, chlorogenic acid, caffeic acid, *p*-coumaric acid, and ferulic acid content in the samples, are presented in Table 1. Compared to RB and RWB, the gallic acid and *p*-coumaric acid content increased (*p* < 0.05), while the protocatechuic acid, chlorogenic acid, caffeic acid, and ferulic acid content decreased (*p* < 0.05) in the sourdough samples. During sourdough fermentation, phenolic acids may be released from their bound state and some converted into intermediates of higher biological activity by LAB and/or yeast [37]. For instance, the decreased ferulic acid content in LAB-fermented substrates was attributed to its degradation into an intermediate such as 4-vinyl guaiacol [38]. Therefore, changes in TPC, TFC, and phenolic acids after sourdough fermentation of substrates may enhance the bioactive potential of the sourdough and resulted sourdough bread [27]. 

Furthermore, the dietary fiber composition of the sourdough samples was presented in Table 1. The results showed that compared to RB and RWB, soluble dietary fiber (SDF) increased (*p* < 0.05), while the insoluble dietary fiber (IDF) and total dietary fiber (TDF) content decreased (*p* < 0.05) in all samples after sourdough fermentation (Table 1). Moreover, a higher increase (*p* < 0.05) in SDF (RWBYK (+48.50%) > RBYK (+47.77%) > RWBY (+26.18%) > RBYK (+25.10%)) and decrease (*p* < 0.05) in TDF (RWBYK (−17.47%) < RBYK (−16.24%) < RWBY (−14.22%) < RBY (−6.01%)) was observed in mixed-strain- than single-strain-fermented red bean with wheat bran than without sourdough. Due to the higher β-glucosidase activity in mixed-strain than single-strain-fermented substrates (Figure 1a), a significantly higher degradation of IDF during sourdough fermentation into its soluble form (SDF) in the fermented substrates may have occurred [38,39]. Similar findings were reported in a recent study where in situ-produced β-glucosidase increased the SDF content and decreased the TDF and IDF in LAB-fermented black bean with added wheat bran [2]. Moreover, the addition of wheat bran to red bean contributed to the increased (*p* < 0.05) TDF content which was increasingly solubilized into more SDF in RWBYK and RWBY relative to RWB than in RBYK and RBY relative to RB (Table 1). 

### 3.3. Baking Characteristics of Bread Prepared with Single- or Mixed-Strain-Fermented Red Bean Supplemented with Wheat Bran Sourdough

#### 3.3.1. Specific Volume and Textural Profile Characteristics of Bread

The effect of incorporation sourdoughs on the specific volume and textural profile characteristics of bread is presented in Table 2. The results showed that compared to WB bread, the addition of red bean flour with wheat bran (RWB) or red bean flour (RB) decreased (*p* < 0.05) the specific volume by −22.89% and −25.63%, and increased (*p* < 0.05) the crumb firmness and chewiness by 97.58% and 102.56%, and 114.12% and 149.77%, respectively. On the other hand, compared to RB and RWB bread controls, incorporation of sourdough increased the specific volume and decreased (*p* < 0.05) the crumb firmness and chewiness of bread. A higher increase (*p* < 0.05) in specific volume (RBYK: +13.86%, RWBYK: +11.99%, RBY: +9.04%, RWBY: +7.62%) and higher decrease (p < 0.05) in crumb firmness (RWBYK: −19.87%, RBYK: −19.31%, RWBY: −14.87%, RBY: −12.78%) and chewiness (RBYK: −31.51%, RWBYK: −26.05%, RBY: −21.59%, RWBY: −17.08%) was observed in mixed-strain- than single-strain-fermented substrates relative to the control breads (Table 2). Given that decreased specific volume coupled with increased crumb firmness and chewiness are known indicators of decreased quality of bread [3,40], these results suggested that the addition of unfermented red bean flour with or without wheat bran was detrimental to the quality of bread, while sourdough fermentation (mixed-strain better than single-strain) of red bean flour with or without wheat bran substrates improved the quality of bread. In earlier studies, phenolic acids and SDF were reported to form networks with gluten protein which resulted in improved structural integrity of dough and gas retention coefficient and enhanced the quality of bread [27,41]. Therefore, the increase in bioactive components such as TPC, TFC, phenolic acids, and SDF in sourdoughs, especially in mixed-strain- followed by single-strain-fermented sourdoughs could have positively contributed to enhanced bread quality. 

#### 3.3.2. Sensory Evaluation of Bread

The results of the sensory evaluation of bread incorporated with single- or mixed-strain-fermented red bean flour with or without wheat bran sourdough are presented in Table 2. The WB bread sample scored highest (*p* < 0.05), while RB followed by RWB scored lowest (*p* < 0.05) in all sensory attributes evaluated. Compared to RB and RWB, all attributes scored higher (*p* < 0.05) in breads prepared with sourdoughs. For instance, the overall acceptance and appearance was rated highest in RWBYK, then RWBY, RBYK, RBY, RB, and RWB; aroma was rated highest in RBYK, then RBY, RWBYK, RWBY, RB, and RWB; taste was rated highest in RBYK, then RWBYK, RBY, RWBY, RB, and RWB; color was rated highest in RWBYK, then RBY, RB, RBYK, RWBY, and RWB; and texture was rated highest in RWBYK, then RBYK, RWBY, RBY, RB, and RWB. These results suggested that sourdough fermentation of substrates, especially mixed-strain then single-strain fermentation, improved several sensory attributes of the resulted bread. This was attributed to the ability of sourdough fermentation to reduce content of ANFs, increase SDF, and release other bioactive compounds such as organic acids, and volatile aromatic compounds, which collectively improved the quality, taste, and aroma of the resulted bread [3]. Therefore, mixed-strain then single-strain sourdough fermentation significantly enhanced the applicability and potential consumer acceptance of red bean flour and wheat bran ingredients in bread [20,21]. 

### 3.4. Effect of Intake of Customized Sourdough Bread Diets on Biochemical Parameters of Serum Samples of Different Mice Groups 

#### 3.4.1. Oral Glucose Tolerance Test (OGTT) and Homeostasis Model Assessment-Insulin Resistance (HOMA-IR) of Mice

**Figure 2:**
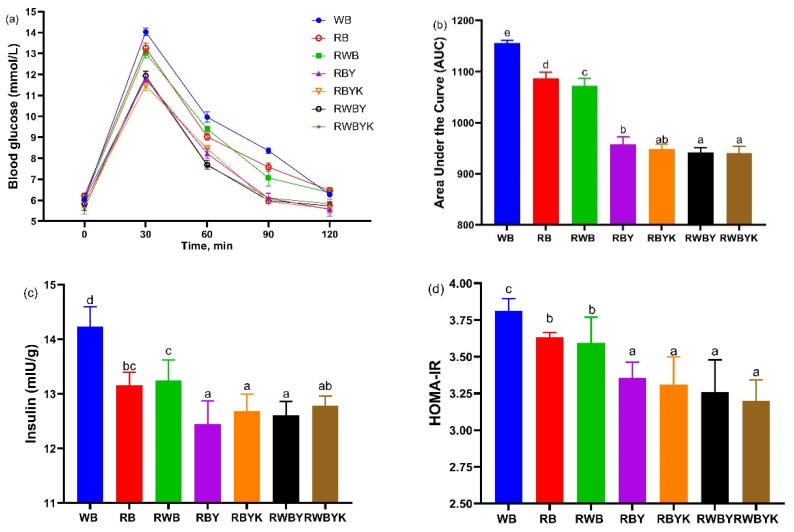
Effect of single- and mixed-strain-fermented red bean flour with or without wheat bran in bread diets on (**a**) oral glucose tolerance test, (**b**) average area under the curve (AUC), (**c**) fasting plasma insulin, and (**d**) homeostasis model assessment-insulin resistance (HOMA-IR) test of mice. The bars represented the mean ± SEM (*n* = 6), with the different letters signifying difference at *p* < 0.05. WB: wheat bread; RB: red bean flour bread; RWB: red bean–wheat bran bread; RBY: red bean sourdough fermented by *L. fermentum* bread; RBYK: red bean sourdough fermented by *L. fermentum* and *K. marxianus* bread. RWBY: red bean–wheat bran sourdough fermented by *L. fermentum* bread. RWBYK: red bean–wheat bran sourdough fermented by *L. fermentum* and *K. marxianus* bread.

The effect of intake of customized sourdough bread diets on blood glucose homeostasis in term of the OGTT and HOMA-IR is shown in Figure 2. The OGTT results revealed that no significant difference (*p* = 0.05) in blood glucose levels was observed at the baseline (0 min) in all mice fed on bread diets (Figure 2a). After injection of glucose, a similar pattern in blood glucose levels, characterized by an increase in the first 30 min, then a rapid decrease from 30 to 60 min, and a leveling off was seen between 60 and 90 min, and 90 and 120 min in all mice groups (Figure 2a). However, the area under the curve (AUC) was highest in the WB group, but decreased (*p* < 0.05) most in mice fed on bread prepared with red bean supplemented with wheat bran sourdough (RWBYK: −17.52% < RWBY: −17.46%), followed by red bean sourdough (RBYK: −16.92% < RBY: −16.10%) and least in unfermented controls (RWB: −6.23% < RB: −5.13%) (Figure 2b). This difference might be associated with their corresponding bioactive components such as TPC, TFC, gallic acid; SDF content in sample RWBYK, RWBY, and RBYK, then RBY was higher in these compounds (Table 1). Meanwhile, this study also revealed that the content TPC, TFC, gallic acid, and SDF were negatively correlated with AUC of mice (Figure S1), indicating that their increased content in bread could improve glucose tolerance in mice (Figure 2a,b). 

Meanwhile, the results of insulin and HOMA-IR in this study revealed that, compared to WB-fed mice, the levels of insulin and HOMA-IR were significantly decreased (*p* < 0.05) after intake of bread diets prepared with unfermented red bean with wheat bran (RWB) or without wheat bran (RB). A similar trend to RWB and RB mice was also noted following intervention of bread diets prepared with single (RBY, RWBY) and mixed (RBYK, RWBYK) strain-fermented sourdoughs (Figure 2c,d). Published studies have reported that intake of breads enriched with dietary fiber and functional ingredients such as phenolic acids had an insulin-resistance-lowering effect [42]. More importantly, a lower decline (*p* = 0.05) in HOMA-IR was observed in mice fed on diets prepared with mixed (RWBYK: −10.62% < RBYK: −8.92%) than single-strain (RWBY: −8.93% < RBY: −7.71%) fermented sourdoughs, but this difference was not observed in RB- and RWB-fed mice (Figure 2d). Here, the HOMA-IR of mice negatively correlated with SDF, gallic acid (*p* < 0.01), TPC (*p* < 0.05), and TFC (*p* < 0.05) content of sourdough used to prepare the bread diets (Figure S1). This indicated that the increased bioactive content in bread due to incorporation of SDF and phenolic-compound-enriched sourdough (especially mixed-strain followed by single-strain) in fermented substrates (especially red bean with wheat bran followed by red bean alone) compared to their unfermented substrates improved insulin sensitivity in healthy mice. Moreover, the positive correlation (*p* < 0.001) between HOMA-IR and AUC in mice further confirmed their interrelatedness in the glucose metabolism of mice (Figure S1) [43].

#### 3.4.2. Lipid Profile in Serum of Mice among Different Groups

In this study, the content of total cholesterol (TC), triglyceride (TG), high-density lipoprotein cholesterol (HDL), and low-density lipoprotein cholesterol (LDL) in serum of mice are presented in Table 3. The results revealed that compared to WB mice, RB- and RWB-fed mice had a decrease (*p* < 0.05) in TC, TG, and LDL, and an increase (*p* < 0.05) in HDL and HDL/LDL ratio. On the other hand, compared to RB- and RWB-fed mice, significantly lower (*p* < 0.05) content of TC, TC, and LDL and higher (*p* < 0.05) content of HDL and HDL/LDL was observed in mice fed on bread diets containing sourdough. Considering that increased levels in TC, TG, and LDL, and decreased HDL levels in serum are associated with the occurrence of insulin resistance [44], the current results might imply that the consumption of bread diets prepared with these substrates (red bean with or without wheat bran) and their sourdough-fermented counterparts improved the serum lipid profile of mice, followed by a subsequently improved insulin resistance. Importantly, a greater outcome was observed in mixed- rather than single-strain-fermented substrates, especially in red bean flour with wheat bran (Table 3). In earlier studies, the mechanism associated with the lipid-lowering effect of intake of bioactive-enriched fermented foods was linked to the activation of fatty acid oxidation of mitochondria and peroxisomes and inhibition of expression of lipogenesis genes in the liver of the host [45]. In this study, the ratio of HDL to LDL was negatively correlated with either HOMA-IR (*p* < 0.001) or AUC (*p* < 0.001) in mice, but it positively correlated with gallic acid (*p* < 0.01), TPC (*p* < 0.05), TFC (*p* < 0.05), and SDF content in sourdoughs (Figure S1). Therefore, it further evidenced that an increased content of SDF and phenolic acids in functionalized wheat bran-based diets positively enhanced healthy benefits in mice [46]. Therefore, we proposed that the diet of RWBYK, RBYK, RWBY, and RBY with a higher bioactive component provides a greater regulation of lipid metabolism and improved lipid profile in serum than the others.

#### 3.4.3. Pro-Inflammatory Cytokine Content in Serum of Mice among Different Groups

The content of pro-inflammatory cytokines, including IL-1β, IL-6, and TNF-α and LPS in serum of mice, is presented in Table 3. The results indicated that, compared to WB, a decrease (*p* < 0.05) in all the parameters was seen in mice fed on RWB, RB, RBY, RWBY, RBYK, and RWBYK bread diets. Moreover, compared to RB- and RWB-fed mice, IL-1β, IL-6, and TNF-α, and LPS content was generally lower in mice fed on bread prepared with mixed (RWBYK < RBYK) than single-strain (RWBY < RBY) fermented red bean flour with wheat bran than without sourdoughs. Similar findings were reported by Chen et al. [6] who found that intake of mixed-strain-fermented white kidney bean sourdough bread significantly reduced the serum content of pro-inflammatory cytokine in healthy mice. A lower grade chronic inflammation is associated with diet-induced insulin resistance in adipose tissues and activation of nuclear factor kappa B (NF-kB) signaling pathway, and induces a higher level of pro-inflammatory cytokines in serum of mice [47]. In contrast, diets with the richness of dietary fiber and phenolic acids could exhibit inhibitory effects on NF-kB signaling, followed by a suppressed secretion of cytokines [47,48,49]. Importantly, the current study revealed that the fermentation, particularly via a mixed-strains model, might further enhance the bioavailability of these active compounds, benefiting a greater function regarding the above bio-parameters of the mice than unfermented ones. Therefore, that might be the key reason why the increased SDF and phenolic acids (e.g., gallic acid) in sourdough bread diets, which were negatively correlated with the levels of IL-1β, IL-6, and TNF-α, and LPS in mice (Figure S1), could have a greater reduced production of cytokines in serum through increased inhibition of the NF-kB signaling pathway in mice than other groups. 

### 3.5. Effect of Customized Sourdough Bread Diets on Gut Microbiota of Mice

**Figure 3:**
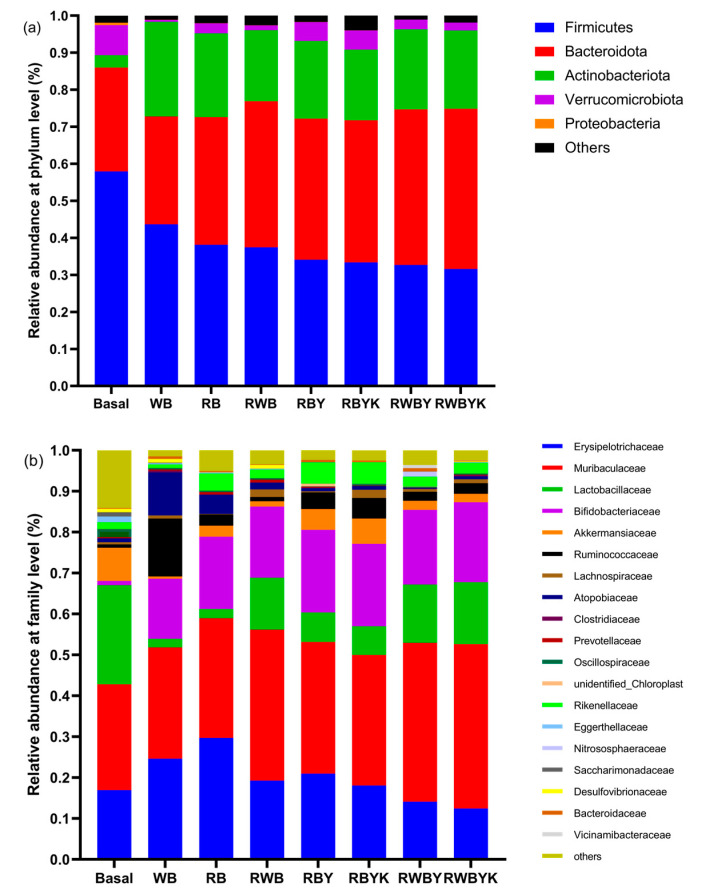

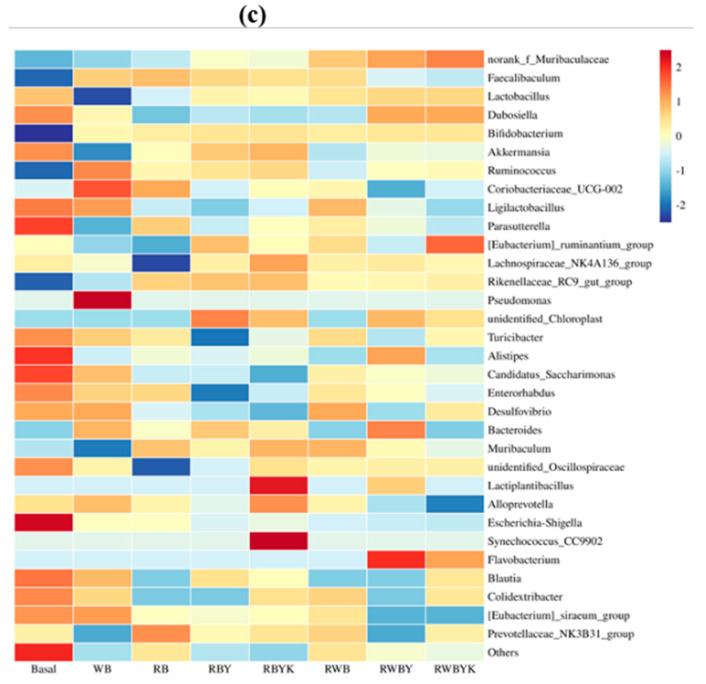
Effect of bread diets on the relative abundance of gut microbiota at, (**a**) the phylum level, (**b**) family level, (**c**) genus level in healthy mice. Basal: mice fed on AIN-93G diet; WB: wheat bread; RB: red bean flour bread; RWB: red bean–wheat bran bread; RBY: red bean sourdough fermented by *L. fermentum* bread; RBYK: red bean sourdough fermented by *L. fermentum* and *K. marxianus* bread; RWBY: red bean–wheat bran sourdough fermented by *L. fermentum* bread; RWBYK: red bean–wheat bran sourdough fermented by *L. fermentum* and *K. marxianus* bread.

Due to the important role played by diet on gut microbiota composition, we studied the effect of bread diets prepared with single- or mixed-strain-fermented red bean flour with or without wheat bran on the gut microbiota of mice. The results presented in Figure 3 show that at the phylum level, *Firmicutes* and *Bacteroidetes* were most abundant in basal mice (85.98%), but declined in WB (72.81%), RB (72.58%), RWB (76.85%), RBY (72.19%), RBYK (71.72%), RWBY (74.69%), and RWBYK (74.85%) fed mice (Figure 3a). Compared to basal mice, *Firmicutes* and *Bacteroidetes* abundance decreased and increased, respectively; moreover, a higher decrease and increase were observed in RWBYK and RBYK, followed by RWBY and RBY and least in RWB-, RB-, and WB-fed mice. These observations were consistent with the findings by Omedi et al. [27] who reported that intake of bread decreased the relative abundance of the dominant phyla *Firmicutes* and *Bacteroidetes*. Furthermore, the abundance of *Actinobacteriota* increased in all mice fed on bread diets. At the family level, compared to the basal group (*Muribaculaceae*: 25.87%, *Lactobacillaceae*: 24.21%, *Erysipelotrichaceae*: 16.79%, *Akkermansiaceae*: 8.12%, *Lanchnospiraceae*: 5.74%, *Rikenellaceae*: 1.65%, and *Bifidobacteriaceae*: 1.03%), an increase in the relative abundance of *Erysipelotrichaceae* (except RWBY, RWBYK), *Muribaculaceae* (higher in presence of wheat bran in red bean after mixed-strain than single-strain fermentation), *Bifidobacteriaceae*, *Lanchnospiraceae* (except RB), and *Rikenellaceae* (except WB), and a decrease in the relative abundance of *Lactobacillaceae* (compared to RB and RWB, increased (*p* < 0.05) abundance in bread diets prepared with wheat bran in red bean after mixed-strain than single-strain fermentation) and *Akkermansiaceae* in mice fed on the bread diets (Figure 3b). As shown in Figure S2, although intake of WB (−1.12%) reduced the α-diversity of gut microbiota, intake of bread prepared with red bean with wheat bran with/out sourdough fermentation (RWBY: +6.76%, RWBYK: +6.62%, RWB: +5.01%) then red bean with/out sourdough fermentation (RBY: +2.81%, RBYK: +3.01%, RB: +1.16%) improved (*p* < 0.05) the α-diversity of gut microbiota of mice relative to basal mice. Furthermore, the heatmap analysis of gut microbiota at the genus level for the 29 most abundant flora revealed significant differences in the different mice groups (Figure 3c). For instance, compared to the basal mice, intake of bread elevated (*p* ≤ 0.001) the abundance of *norank_f_Muribaculaceae*, *Lactobacillus* (higher abundance in RWBYK and RWBY, then RWB, RBYK and RBY, RB, WB), *Akkermansia*, *Muribaculum*, *Rikenellaceae* RC9 gut group, *Bifidobacterium* (higher in mixed-fermented red bean and wheat bran) in mice. However, the abundance of *Faecalibaculum*, *Dubosiella* (except in RWBYK and RWBY), and pathogenic microbiota *Escherichia Shigella*, *Desulfovibrio,* and *Candidatus_Saccharimonas* decreased after intake of the experimental bread diets in mice (Figure 3c). The changes in gut microbiota composition may be attributed to increased bioactive components such as SDF and phenolic acids in the bread diets fed to mice. In earlier studies, dietary fibers and phenolic acids were found to selectively increase bacterial diversity, and enrichment of beneficial gut microbiota [50,51,52]. As shown in Figure S1, an increased abundance of *Bacteroidetes*, *Muribaculaceae*, *Bifidobacteriaceae*, and *Lactobacillus* was positively associated with TFC, TPC, gallic acid, SDF of sourdoughs, and α-diversity of gut microbiota, but negatively associated with serum pro-inflammatory cytokines (IL-6, TNF-α, IL-1β), LPS, AUC of OGTT, HOMA-IR, TC, LDL, and TG of mice. Therefore, the current study further supported that the enrichment of those active compound in the diet benefited the promotion of beneficial bacteria in terms of *Bifidobacteriaceae* and *Lactobacillus*, following their positive impact on serum bio-parameters as mentioned above. On the other hand, an opposite trend in correlation was also seen in *Firmicutes* and *Actinobacteriota* in mice (Figure S1). Closely comparable observations were made in high-fat-induced obesity mice fed on insoluble yeast β-glucan [53]. Therefore, the prebiotic effect of bread diets attributed to mixed- and single-strain sourdough fermentation of red bean with or without wheat bran which increased the content of bioactive and potential prebiotic compounds such as SDF and phenolic acids in bread diets increased gut microbiota diversity and the relative abundance of highly probiotic and fermentative microbiota in mice. 

### 3.6. Effect of Customized Sourdough Bread Diets on Short-Chain Fatty Acids (SCFAs) in Mice

**Figure 4:**
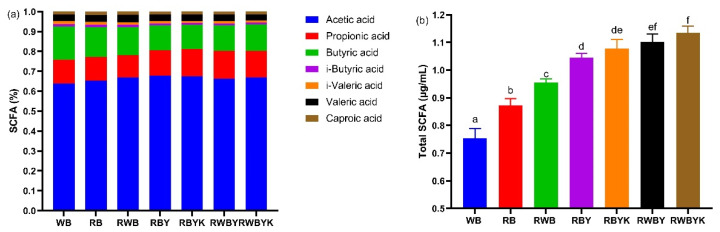
Individual short-chain fatty acids (SCFAs) content (**a**) and total SCFA content (**b**) in the feces of the mice following different diet intervention. The bars represented the mean ± SEM (*n* = 3), with the different letters signifying difference at *p* < 0.05. WB: wheat bread; RB: red bean flour bread; RWB: red bean–wheat bran bread; RBY: red bean sourdough fermented by *L. fermentum* bread; RBYK: red bean sourdough fermented by *L. fermentum* and *K. marxianus* bread; RWBY: red bean–wheat bran sourdough fermented by *L. fermentum* bread; RWBYK: red bean–wheat bran sourdough fermented by *L. fermentum* and *K. marxianus* bread.

To establish the effect of bread diets on the key microbial metabolites, the content of SCFA in mice was analyzed and the results are presented in Figure 4. Acetic acid had the highest level (0.273–0.454 µg/mL; 63.83–67.82%), followed by butyric acid (0.152–0.199 µg/mL; 12.06–16.62%) and propionic acid (0.099–0.168 µg/mL; 11.11–13.99%). Meanwhile, other acidic compounds were also measured, such as valeric acid (0.084–0.137 µg/mL; 3.15–3.82%), caproic acid (0.026–0.034 µg/mL; 1.36–1.62%), iso-valeric acid (0.058–0.065 µg/mL; 0.98–1.55%), and iso-butyric acid (0.026–0.034 µg/mL; 0.91–1.30%) (Figure 4a). Total SCFA was highest (*p* < 0.05) in RWBYK, followed by RWBY, then RBYK, RBY, RWB, and RB, and least in WB among all the mice groups (Figure 4b). These observations suggested that sourdough fermentation of red bean with wheat bran then red bean alone significantly increased production of microbial SCFA in mice. This may be attributed to a dual-interaction model, in which the diet of either RWBYK or RWBY was characterized with a higher level of TPC, TFC, phenolic acids, and SDF in sourdoughs, delivering into the gut for selectively promoting the growth of *Bifidobacteriaceae* and *Lactobacillus*, followed by a depressed generation of pathogenic bacterial *Escherichia Shigella* and *Desulfovibrio*. On the other hand, the shift of the gut microbiota profile induced an increased and quicker generation of SCFAs, which could play the role of a depressor of pathogenic growth. The association analysis further supported this proposed mechanism, in which total SCFAs were positively correlated with TPC, gallic acid, TFC, HDL/LDL, SDF, and α-diversity of mice, but negatively correlated with pro-inflammatory cytokines (IL-1β, IL-6, TNF-α), LPS, AUC, and HOMA-IR of mice (Figure S1). These correlations implied that the microbial SCFA produced due to the prebiotic effect of bread diets enhanced gut microbiota diversity and promoted health benefits such as anti-inflammation effects, improved insulin sensitivity, and glucose tolerance in the mice [6,20,54,55]. Therefore, mixed-strain then single-strain sourdough fermentation of red bean with wheat bran significantly enhanced the prebiotic effect of the resulted sourdough bread diets. The increased prebiotic effect increased gut microbiota diversity and positively influenced the release of SCFAs which exhibited the health benefits in these mice. 

## 4. Conclusions

**Figure 5:**
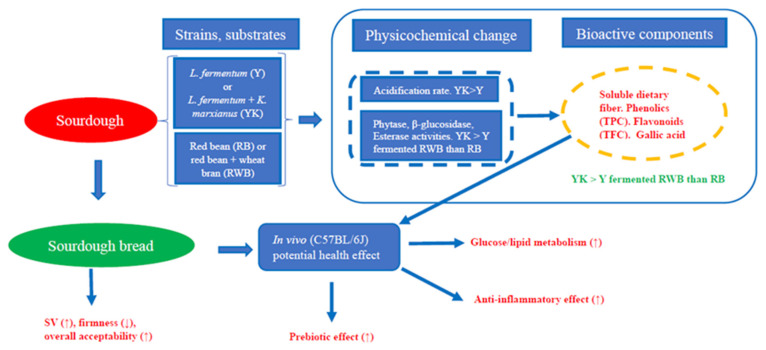
A schematic diagram showing a regulation model via different diet interventions in mice. Y: single (*L. fermentum*) strain fermentation; YK: mixed (*L. fermentum* and *K. marxianus*) strain fermentation. (↑) and (↓) represented an increase and decrease in the specific parameter, respectively. RB: red bean substrate; RWB: red bean with wheat bran substrate; RB: red bean flour bread; RWB: red bean–wheat bran bread; RBY: red bean sourdough fermented by *L. fermentum* bread; RBYK: red bean sourdough fermented by *L. fermentum* and *K. marxianus* bread; RWBY: red bean–wheat bran sourdough fermented by *L. fermentum* bread; RWBYK: red bean–wheat bran sourdough fermented by *L. fermentum* and *K. marxianus* bread.

In conclusion, compared to a single-strain fermentation, the sourdough prepared via a mixed-strain fermentation of red bean with/out wheat bran had greater decreased anti-nutritional factors and a higher increased bioactive component in substrates. Significantly higher content of soluble dietary fiber, total phenols and flavonoid, and phenolic acids such as gallic acid were obtained after the mixed-strain fermentation than single-strain sourdough fermentation. Subsequently, although the quality and sensory acceptance of breads were greatly improved via either single- or mixed-strain fermentation, mixed-strain fermentation could better improve the quality of the bread compared to single-strain fermentation. Furthermore, intake of sourdough-based breads lowered serum pro-inflammatory cytokine content, and improved serum lipid profile, glucose tolerance, and insulin sensitivity in mice. In addition, sourdough-based bread diets increased gut microbiota diversity towards beneficial genera (e.g., *Lactobacillus*, *Bifidobacterium*, *Muribaculaceae*) while suppressing harmful genera (e.g., *Escherichia Shigella*, *Desulfovibrio*), accompanied with increased production of short-chain fatty acids in mice. Overall, better outcomes were found in bread prepared with mixed- then single-strain-fermented red bean with wheat bran sourdough than the sourdough without wheat bran. These results suggested that in situ-produced enzymes and acidification during mixed- then single-strain substrate fermentation increased the bioactive content and prebiotic effect of the sourdough-based bread diets. In conclusion, the synergy during mixed- rather than single-strain fermentation of high-fiber-legume flour baked products could improve the baking quality and sensory quality, and enhance the prebiotic effect of bread leading to increased potential health benefits in mice. Based on the current study, a regulation model via different diet interventions is proposed in Figure 5.

## Figures and Tables

**Table 1:** Physicochemical characteristics of red bean flour with or without wheat bran sourdough fermented by single (*L. fermentum*) or mixed (*L. fermentum* and *K. marxianus*) strain.

Item		RB	RWB	RBY	RBYK	RWBY	RWBYK
**Physicochemical characteristics**	pH	6.46 ± 0.03 ^e^	6.39 ± 0.06 ^d^	4.37 ± 0.03 ^c^	4.27 ± 0.02 ^ab^	4.31 ± 0.04 ^bc^	4.23 ± 0.02 ^a^
	TTA (mL)	4.52 ± 0.03 ^a^	4.48 ± 0.07 ^a^	18.28 ± 0.15 ^b^	19.99 ± 0.14 ^d^	19.27 ± 0.09 ^c^	20.39 ± 0.07 ^e^
**Anti-nutritional factors**	Condensed tannin (mg/g)	6.14 ± 0.34 ^c^	3.59 ± 0.22 ^b^	3.61 ± 0.31 ^b^	3.68 ± 0.19 ^b^	2.47 ± 0.26 ^a^	2.43 ± 0.12 ^a^
	Phytic acid (mg/g)	6.54 ± 0.28 ^b^	11.65 ± 0.25 ^d^	5.09 ± 0.09 ^a^	4.98 ± 0.06 ^a^	8.22 ± 0.14 ^c^	8.14 ± 0.14 ^c^
	Trypsin inhibitor (mg/g)	13.95 ± 0.21 ^d^	9.34 ± 0.25 ^c^	8.14 ± 0.13 ^b^	8.20 ± 0.14 ^b^	5.05 ± 0.07 ^c^	5.12 ± 0.11 ^c^
**Phytochemical content**	TPC (mg GAE/g)	4.02 ± 0.08 ^a^	4.95 ± 0.08 ^b^	5.24 ± 0.12 ^c^	5.60 ± 0.12 ^d^	6.63 ± 0.13 ^e^	7.18 ± 0.14 ^f^
	TFC (mg RE/g)	2.39 ± 0.10 ^a^	2.31 ± 0.16 ^a^	2.94 ± 0.14 ^b^	3.32 ± 0.13 ^c^	2.82 ± 0.15 ^b^	3.17 ± 0.11 ^c^
**Phenolic acids (µg/g)**	Gallic acid	47.74 ± 3.27 ^a^	54.74 ± 4.78 ^ab^	59.67 ± 4.28 ^bc^	68.19 ± 5.28 ^de^	64.72 ± 3.84 ^cd^	73.12 ± 4.41 ^e^
	Protocatechuic acid	73.26 ± 2.22 ^f^	60.80 ± 3.67 ^e^	35.18 ± 2.58 ^d^	19.07 ± 2.56 ^a^	28.02 ± 2.05 ^c^	20.35 ± 2.33 ^b^
	Chlorogenic acid	28.63 ± 2.60 ^d^	15.19 ± 0.75 ^b^	14.73 ± 0.53 ^b^	17.83 ± 0.96 ^c^	4.49 ± 0.40 ^a^	4.32 ± 0.49 ^a^
	Caffeic acid	17.06 ± 1.11 ^d^	8.90 ± 0.41 ^bc^	8.65 ± 0.51 ^b^	9.92 ± 0.49 ^c^	4.51 ± 0.48 ^a^	4.09 ± 0.43 ^a^
	*p*-Coumaric acid	-	0.54 ± 0.12 ^c^	0.18 ± 0.12 ^a^	0.26 ± 0.09 ^b^	1.23 ± 0.10 ^e^	0.92 ± 0.19 ^d^
	Ferulic acid	11.18 ± 0.32 ^d^	16.55 ± 1.07 ^e^	8.82 ± 0.39 ^a^	8.98 ± 0.34 ^b^	10.34 ± 0.45 ^cd^	10.11 ± 0.40 ^c^
**Dietary fiber (g/100 g)**	IDF	4.23 ± 0.07 ^c^	7.26 ± 0.09 ^f^	3.72 ± 0.08 ^b^	3.01 ± 0.05 ^a^	5.60 ± 0.08 ^e^	4.97 ± 0.07 ^d^
	SDF	0.82 ± 0.03 ^a^	1.55 ± 0.04 ^d^	1.03 ± 0.05 ^b^	1.22 ± 0.02 ^c^	1.96 ± 0.03 ^e^	2.31 ± 0.04 ^f^
	TDF	5.05 ± 0.04 ^c^	8.81 ± 0.14 ^f^	4.75 ± 0.09 ^b^	4.23 ± 0.07 ^a^	7.56 ± 0.06 ^e^	7.27 ± 0.08 ^d^

Data are presented as the mean ± SD (*n* = 3) with different letters in the same row indicating a significant difference at *p* < 0.05 (Duncan’ test). (-) indicates not determined. RB: red bean flour slurry (DY: 300); RWB: red bean–wheat bran slurry (red bean to wheat bran, 5:4, (*w*/*w*)) (DY: 300); RBY: red bean sourdough fermented by *L. fermentum*; RBYK: red bean sourdough fermented by *L. fermentum* and *K. marxianus*. RWBY: red bean–wheat bran sourdough fermented by *L. fermentum*. RWBYK: red bean–wheat bran sourdough fermented by *L. fermentum* and *K. marxianus*. TT: total titratable acidity. TPC: total phenolic compound. GAE: gallic acid equivalent. TFC: total flavonoid content. RE: rutin equivalent. IDF: insoluble dietary fiber. SDF: soluble dietary fiber. TDF: total dietary fiber.

**Table 2:** Effect of single- and mixed-strain-fermented red bean flour with or without wheat bran sourdough on the specific volume, textural profile characteristics, and sensory evaluation of wheat bread.

	Specific Volume (mL/g)	Textural Profile Characteristics		Sensory Evaluation					
Sample (s)		Firmess (g)	Chewiness (mJ)	Texture	Taste	Color	Aroma	Appearance	Overall Acceptability
WB	6.08 ± 0.14 ^e^	234.33 ± 8.14 ^a^	14.40 ± 0.66 ^a^	7.35 ± 0.49 ^c^	7.50 ± 0.71 ^d^	6.95 ± 0.21 ^b^	7.05 ± 0.14 ^d^	7.65 ± 0.35 ^c^	7.70 ± 0.14 ^d^
RB	4.52 ± 0.17 ^a^	474.67 ± 5.03 ^f^	35.97 ± 1.59 ^f^	5.70 ± 0.28 ^a^	6.30 ± 0.14 ^ab^	6.75 ± 0.07 ^ab^	6.45 ± 0.14 ^b^	6.60 ± 0.14 ^ab^	5.95 ± 0.24 ^a^
RWB	4.69 ± 0.08 ^a^	463.00 ± 9.16 ^e^	30.83 ± 1.17 ^e^	5.40 ± 0.14 ^a^	6.15 ± 0.07 ^a^	6.55 ± 0.14 ^a^	6.00 ± 0.13 ^a^	6.35 ± 0.07 ^a^	5.65 ± 0.21 ^a^
RBY	4.93 ± 0.09 ^b^	414.00 ± 6.08 ^d^	28.20 ± 1.18 ^d^	6.50 ± 0.14 ^b^	6.85 ± 0.14 ^abcd^	6.80 ± 0.07 ^ab^	6.95 ± 0.07 ^d^	6.60 ± 0.21 ^ab^	6.80 ± 0.07 ^b^
RBYK	5.15 ± 0.02 ^cd^	383.00 ± 6.24 ^c^	24.63 ± 0.87 ^bc^	6.75 ± 0.06 ^b^	7.05 ± 0.08 ^cd^	6.75 ± 0.01 ^ab^	7.10 ± 0.01 ^d^	6.90 ± 0.01 ^bc^	6.90 ± 0.14 ^b^
RWBY	5.04 ± 0.04 ^bc^	393.67 ± 4.51 ^c^	25.57 ± 0.65 ^c^	6.60 ± 0.03 ^b^	6.70 ± 0.01 ^abc^	6.70 ± 0.07 ^ab^	6.60 ± 0.06 ^bc^	6.90 ± 0.14 ^bc^	7.00 ± 0.07 ^bc^
RWBYK	5.25 ± 0.04 ^d^	371.33 ± 5.20 ^b^	22.80 ± 0.89 ^b^	6.85 ± 0.04 ^bc^	7.00 ± 0.07 ^bcd^	6.85 ± 0.07 ^b^	6.80 ± 0.21 ^cd^	7.20 ± 0.28 ^c^	7.30 ± 0.06 ^c^

Data are presented as the mean ± SD (*n* = 3) with different letters in the same column indicating significant difference at *p* < 0.05 (Duncan’s test). WB: wheat bread; RB: red bean flour bread; RWB: red bean–wheat bran bread; RBY: red bean sourdough fermented by *L. fermentum* bread; RBYK: red bean sourdough fermented by *L. fermentum* and *K. marxianus* bread. RWBY: red bean–wheat bran sourdough fermented by *L. fermentum* bread. RWBYK: red bean–wheat bran sourdough fermented by *L. fermentum* and *K. marxianus* bread.

**Table 3:** Effect of single- and mixed-strain-fermented red bean flour with or without wheat bran sourdough in bread diets on serum lipid profile and cytokine content in healthy mice.

	Lipid Profile (mmol/L)					Cytokine Content			
Sample (s)	TC	TG	HDL	LDL	HDL:LDL	IL-1β (pg/L)	IL-6 (pg/L)	TNF-α (ng/L)	LPS (ng/L)
WB	3.64 ± 0.06 ^c^	0.20 ± 0.00 ^b^	1.21 ± 0.02 ^a^	2.28 ± 0.02 ^c^	0.53 ± 0.01 ^a^	116.10 ± 0.56 ^d^	123.98 ± 1.89 ^d^	564.69 ± 5.05 ^d^	341.24 ± 5.63 ^e^
RB	3.57 ± 0.05 ^b^	0.20 ± 0.00 ^b^	1.23 ± 0.01 ^b^	2.28 ± 0.02 ^c^	0.54 ± 0.01 ^ab^	114.02 ± 1.24 ^c^	119.45 ± 1.45 ^c^	548.40 ± 3.55 ^c^	314.33 ± 2.96 ^d^
RWB	3.56 ± 0.04 ^b^	0.20 ± 0.00 ^b^	1.24 ± 0.01 ^bc^	2.27 ± 0.02 ^c^	0.55 ± 0.00 ^b^	114.46 ± 0.96 ^c^	118.83 ± 1.41 ^c^	551.30 ± 4.60 ^c^	309.60 ± 3.40 ^c^
RBY	3.41 ± 0.06 ^a^	0.19 ± 0.00 ^a^	1.25 ± 0.01 ^cd^	2.22 ± 0.02 ^b^	0.56 ± 0.01 ^c^	111.26 ± 0.76 ^b^	116.27 ± 1.60 ^b^	536.40 ± 4.85 ^b^	293.36 ± 2.72 ^ab^
RBYK	3.37 ± 0.04 ^a^	0.19 ± 0.00 ^a^	1.25 ± 0.01 ^cd^	2.20 ± 0.02 ^ab^	0.57 ± 0.01 ^cd^	110.12 ± 1.14 ^ab^	113.52 ± 1.09 ^a^	531.40 ± 3.97 ^b^	291.10 ± 2.81 ^a^
RWBY	3.37 ± 0.02 ^a^	0.19 ± 0.01 ^a^	1.25 ± 0.01 ^cd^	2.21 ± 0.04 ^ab^	0.57 ± 0.01 ^cd^	110.63 ± 0.72 ^b^	115.59 ± 1.77 ^b^	532.49 ± 6.24 ^b^	296.60 ± 2.56 ^b^
RWBYK	3.39 ± 0.06 ^a^	0.19 ± 0.00 ^a^	1.26 ± 0.02 ^d^	2.19 ± 0.02 ^a^	0.57 ± 0.01 ^d^	109.46 ± 1.02 ^a^	111.92 ± 1.38 ^a^	520.20 ± 3.44 ^a^	293.96 ± 3.29 ^ab^

Data are presented as the mean ± SD (*n* = 6) with different letters in the same column indicating significant difference at *p* < 0.05 (Duncan’ test). TC: Total cholesterol; TG: Triglyceride; HDL: High-density lipoprotein cholesterol; LDL: Low-density lipoprotein cholesterol; IL-1β: Interleukin-1 beta; IL-6: Interleukin-6p TNF-α: Tumor necrosis factor-alpha; LPS: Lipopolysaccharide; WB: wheat bread; RB: red bean flour bread; RWB: red bean–wheat bran bread; RBY: red bean sourdough fermented by *L. fermentum* bread; RBYK: red bean sourdough fermented by *L. fermentum* and *K. marxianus* bread; RWBY: red bean–wheat bran sourdough fermented by *L. fermentum* bread; RWBYK: red bean–wheat bran sourdough fermented by *L. fermentum* and *K. marxianus* bread.

## Data Availability

The data presented in this study are available on request from the corresponding authors. The data are not publicly available due to privacy restrictions.

## Supplementary Materials

The following supporting information can be downloaded at: https://www.mdpi.com/article/10.3390/foods13172856/s1, Figure S1: Pearson correlation of bioactive components in bread diets prepared with sourdough on gut microbiota and potential health benefits in healthy mice. Red: positive correlation, blue: negative correlation. * *p* < 0.05, ** *p* < 0.01, *** *p* < 0.001 were represented for significant correlation; Figure S2: Changes in Shannon α-diversity index in mice fed on bread diets prepared with single- and mixed-strain-fermented red bean flour with or without wheat bran sourdough. Data are presented as the mean ± SD (n = 6) with different letters in the same column indicating significant difference at *p* < 0.05 (Duncan’ test). Basal: mice fed on AIN-93G diet. WB: wheat bread; RB: red bean flour bread; RWB: red bean–wheat bran bread; RBY: red bean sourdough fermented by *L. fermentum* bread; RBYK: red bean sourdough fermented by *L. fermentum* and *K. marxianus* bread; RWBY: red bean–wheat bran sourdough fermented by *L. fermentum* bread. RWBYK: red bean–wheat bran sourdough fermented by *L. fermentum* and *K. marxianus* bread; Table S1: The recipe of the different types of breads; Table S2: Formulation and nutrition of the different customized bread diets.
