# Supplementary material for: The Effects of Single- or Mixed-Strain Fermentation of Red Bean Sourdough, with or without Wheat Bran, on Bread Making Performance and Its Potential Health Benefits in Mice Model
Source: Foods. 2024 Sep 9;13(17):2856. doi: 10.3390/foods13172856 (PMC11394683; doi:10.3390/foods13172856)
Supplement: Supplementary file 1 [file foods-13-02856-s001.zip › foods-3131289-supplementary.pdf]

foods

MDPI

Supplementary material

Table S1. The recipe of the different types of breads.

	Sample ID						
**Ingredient (s)**	**WB**	**RB**	**RWB**	**RBY**	**RBYK**	**RWBY**	**RWBYK**
Wheat flour (g)	300	255	255	255	255	255	255
Red bean flour (g)	-	45	25	-	-	-	-
Wheat bran (g)	-	-	20	-	-	-	-
Yeast* (g)	4.5	4.5	4.5	4.5	4.5	4.5	4.5
Sugar (g)	16	16	16	16	16	16	16
Salt (g)	3	3	3	3	3	3	3
Shortening** (g)	12	12	12	12	12	12	12
Sourdough (g)	-	-	-	135	135	135	135
Water (mL)	180	170	170	80	80	80	80

WB: wheat bread; RB: red bean bread; RWB: red bean-wheat bran bread (red bean: wheat bran, 5:4, (w/w)); RBY: red bean sourdough bread fermented by *L. fermentum*; RBYK: red bean sourdough bread fermented by *L. fermentum* and *K. marxianus*. RWBY: red bean-wheat bran sourdough bread fermented by *L. fermentum*. RWBYK: red bean-wheat bran sourdough bread fermented by *L. fermentum* and *K. marxianus*. *Bakers yeast (Saf-instant), ingredients: yeast (*Saccharomyces cerevisiae*), emulsifier (sorbitan monostearate), antioxidant (ascorbic acid). **Shortening (COFCO Donghai Grain and Oil Industry, Zhangjiagan, Co. Ltd), ingredients: refined vegetable oil, refined edible butter, water, food additive (monodiglycerol fatty acid ester, soybean phospholipid, tert butylhydroquinone (TBHQ), β-carotene, citric acid), food essence. Added to enhance softness.

Table S2. Formulation and nutrition of the different customized bread diets.

	Customized bread diets**						
**Ingredient (g)**	**WB**	**RB**	**RWB**	**RBY**	**RBYK**	**RWBY**	**RWBYK**
Bread powder*	500	500	500	500	500	500	500
Casein, 30 Mesh	100	100	100	100	100	100	100
L-Cystine	1.5	1.5	1.5	1.5	1.5	1.5	1.5
Corn Starch	198.5	198.5	198.5	198.5	198.5	198.5	198.5
Maltodextrin	66	66	66	66	66	66	66
Sucrose	50	50	50	50	50	50	50
Cellulose	25	25	25	25	25	25	25
Soybean Oil	35	35	35	35	35	35	35
t-Butylhydroquinone	0.007	0.007	0.007	0.007	0.007	0.007	0.007
Mineral Mix S10022G	17.5	17.5	17.5	17.5	17.5	17.5	17.5
Vitamin Mix V10037	5	5	5	5	5	5	5
Choline Bitartrate	1.25	1.25	1.25	1.25	1.25	1.25	1.25
**Total**	1000	1000	1000	1000	1000	1000	1000

*Bread (Table S1) dried (40 °C for 9 h) and pulverized into a powder. ** Processed into pellets, purified (irradiated) by Anhui Xiaobu Qianli Biotechnology Co., Ltd (Anhui, China).

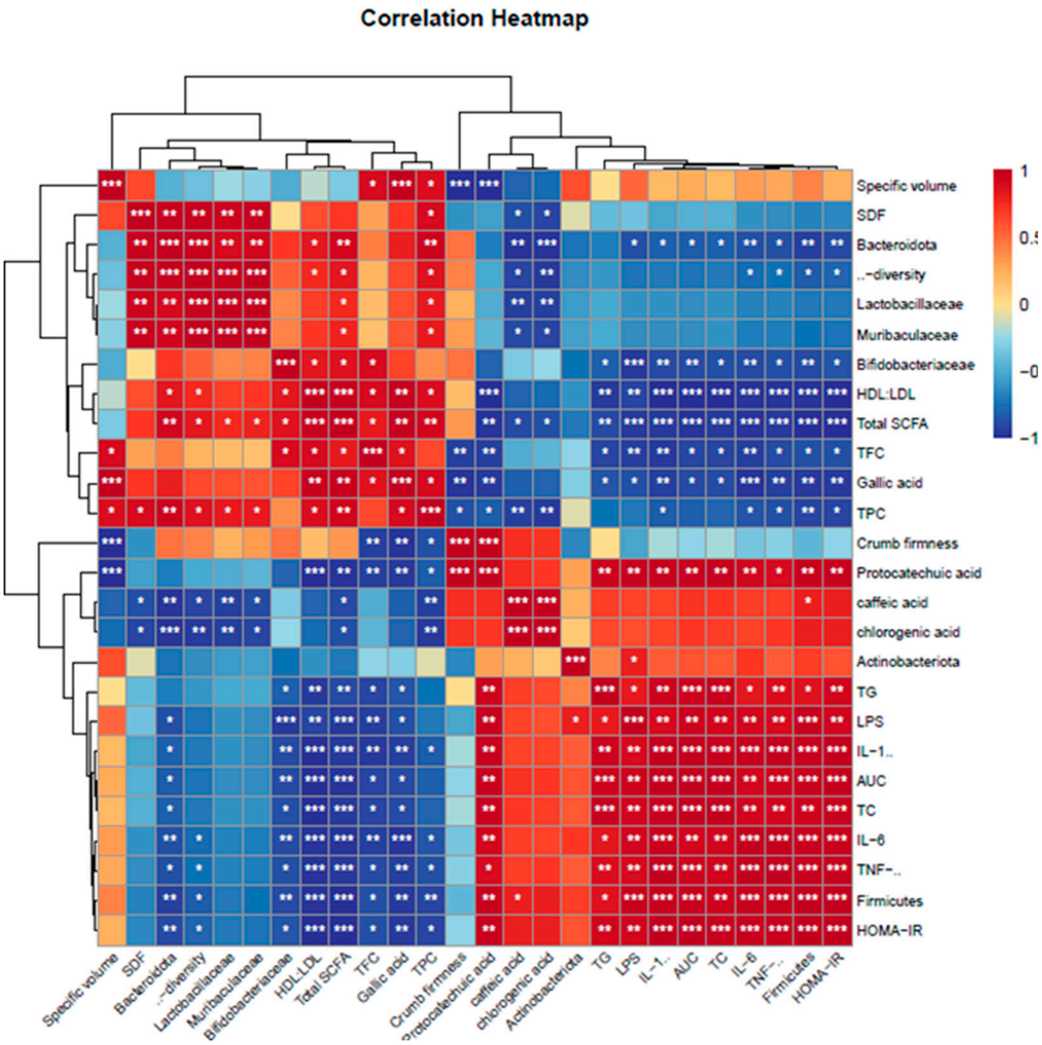

Figure S1. Pearson correlation of bioactive components in bread diets prepared with sourdough on gut microbiota and potential health benefits in healthy mice. Red: positive correlation, blue: negative correlation. *$p < 0.05$, **$p < 0.01$, ***$p < 0.001$ were represented for significant correlation.

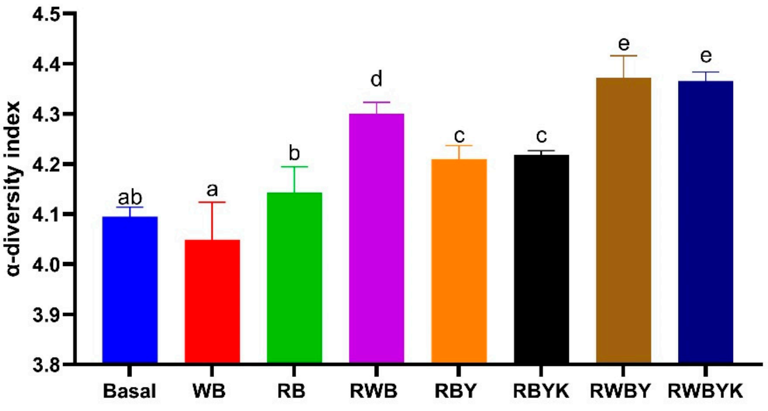

Figure S2. Changes in Shannon α-diversity index in mice fed on bread diets prepared with single and mixed strain fermented red bean flour with or without wheat bran sourdough. Data was presented as the mean ± SD (n = 6) with different letters in the same column indicating significant difference at $p <$ 0.05 (Duncan' test). Basal: mice fed on AIN-93G diet. WB: wheat bread. RB: red bean flour bread; RWB: red bean-wheat bran bread; RBY: red bean sourdough fermented by *L. fermentum* bread; RBYK: red bean sourdough

fermented by *L. fermentum* and *K. marxianus* bread. RWBY: red bean-wheat bran sourdough fermented by *L. fermentum* bread. RWBYK: red bean-wheat bran sourdough fermented by *L. fermentum* and *K. marxianus* bread.
